# Retinal Layer Thickness for Risk Stratification of Progression Independent of Relapse Activity in Relapsing‐Remitting Multiple Sclerosis

**DOI:** 10.1002/brb3.71612

**Published:** 2026-07-28

**Authors:** Andre Braginets, Frederike Cosima Oertel, Eva‐Maria Strauß, Carla Leutloff, Tanja Schmitz‐Hübsch, Friedemann Paul, Susanna Asseyer, Rebecca Wicklein, Bernhard Hemmer, Klemens Ruprecht, Hanna Gwendolyn Zimmermann, Benjamin Knier, Ting‐Yi Lin

**Affiliations:** ^1^ Experimental and Clinical Research Center a Cooperation between Max Delbrück Center for Molecular Medicine in the Helmholtz Association and Charité‐Universitätsmedizin Berlin Berlin Germany; ^2^ NCRC–Neuroscience Clinical Research Center Charité‐Universitätsmedizin Berlin, Corporate Member Of Freie Universität Berlin and Humboldt‐Universität zu Berlin Berlin Germany; ^3^ Department of Neurology Charité‐Universitätsmedizin Berlin, Corporate Member of Freie Universität Berlin and Humboldt‐Universität zu Berlin Berlin Germany; ^4^ Department of Neurology, Klinikum Rechts der Isar, TUM School of Medicine and Health Technical University of Munich Munich Germany; ^5^ Munich Cluster of Systems Neurology (SyNergy) Munich Germany; ^6^ Division of Neuroimmunology, Department of Neurology University of Heidelberg Heidelberg Germany; ^7^ Einstein Center Digital Future Berlin Germany; ^8^ Department of Neurology Diak Klinikum Schwäbisch Hall Schwäbisch Hall Germany; ^9^ Department of Neurology Johns Hopkins University Baltimore Maryland USA

**Keywords:** multiple sclerosis (MS), optical coherence tomography (OCT), progression independent of relapse activity (PIRA), retinal layer thickness

## Abstract

**Background:**

Identifying people with relapsing‐remitting multiple sclerosis (pwRRMS) at risk of progression independent of relapse activity (PIRA) remains an unmet clinical need. Optical coherence tomography (OCT)‐derived measures of retinal neuroaxonal damage may provide prognostic value. This study aimed to evaluate whether peripapillary retinal nerve fiber layer (pRNFL) and ganglion cell‐inner plexiform layer (GCIP) thickness predict PIRA within a heterogenous group of pwRRMS.

**Methods:**

We retrospectively screened pwRRMS with ≥ 2 years relapse‐free follow‐up from prospective observational cohort studies in Berlin (*n* = 74) and Munich (*n* = 146). PIRA was defined as disability worsening, determined by method‐specific thresholds across multimodal assessments, sustained until end of follow‐up. Analyses were restricted to non‐optic neuritis eyes. Cox hazard models were used to assess the adjusted hazard (aHR) of PIRA with age‐adjusted pRNFL or GCIP *Z*‐scores.

**Results:**

Out of 220 pwRRMS (age: 38.1 ± 9.9 years), 65 (29.5%) developed PIRA over a follow‐up of 2.9 [IQR: 1.6–4.1] years. No associations between OCT measures and PIRA were found within the overall study population or Munich cohort. In the Berlin cohort, thinner pRNFL predicted PIRA (aHR [95% CI] = 1.41 [1.03–1.92], *p* = 0.032), while GCIP did not (aHR [95% CI] = 1.38 [0.90–2.10], *p* = 0.140).

**Conclusions:**

OCT measurements do not predict the hazard of PIRA within a heterogeneous group of pwRRMS. However, they have potential prognostic value in early disease stages. Clinical characteristics might influence the association and require further investigation.

## Graphical abstract

This study assessed the prognostic value of optical coherence tomography‐derived thicknesses of the peripapillary retinal nerve fiber and ganglion cell‐inner plexiform layer for identifying people with relapsing‐remitting multiple sclerosis at risk of progression independent of relapse activity (PIRA) from two German centers. Age‐adjusted *Z*‐scores of both layers predicted PIRA in the Berlin cohort but not in Munich or the overall sample, highlighting their potential to identify individuals prone to progression while emphasizing the influence of clinical variability on their applicability.

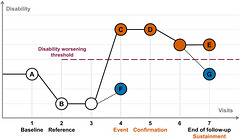

## 1 Introduction

Progression independent of relapse activity (PIRA) is increasingly recognized as a major contributor to disability accrual in people with relapsing‐remitting multiple sclerosis (pwRRMS), challenging the current clinical dichotomy between relapsing and progressive multiple sclerosis (MS) phenotypes (Kappos et al. 2020; Lublin et al. 2014; 2022; Müller et al., 2023). It is suggested to be a clinical representation of an insidious neurodegenerative process occurring from disease onset (Giovannoni et al., 2022; Cagol et al., 2022; 2024; Portaccio et al., 2022; Tur et al., 2023).

Disease progression is clinically assessed in retrospect, leading to a delayed detection and reevaluation of treatment (Lublin et al., 2014; Portaccio et al., 2022). PwRRMS with PIRA have been shown to reach higher levels of disability in shorter time as compared to pwRRMS without PIRA (Tur et al., 2023). These highlights the clinical need for biomarkers that can prospectively identify pwRRMS at risk of developing PIRA.

The Expanded Disability Status Scale (EDSS) is, despite its low intra‐ and inter‐rater reliability and overemphasize on ambulation, the most commonly used clinical measurement to assess disability in pwRRMS (Meyer‐Moock et al., 2014). Composite performance measures incorporating multiple functional domains, including Timed 25‐foot Walk (T25FW), 9‐Hole Peg Test (9‐HPT), and Symbol Digit Modalities Test (SDMT), have demonstrated superior sensitivity in detecting PIRA events (Kappos et al., 2020)

Given the fluctuating nature of MS disability with periods of transient worsening or improvement, the concept of *sustained PIRA* has been proposed to capture persistent deteriorations without recovery, thus identifying clinically meaningful long‐term disability accrual (Lublin et al., 2022; Müller et al., 2023).

Optical coherence tomography (OCT) offers unique insights into MS‐related neurodegeneration through high‐resolution retinal imaging. The peripapillary retinal nerve fiber layer (pRNFL) reflects axonal integrity, while the ganglion cell‐inner plexiform layer (GCIP) represents neuronal health. Multiple studies have shown that pRNFL and GCIP thinning can reflect and predict disability accrual, as well as other disease activity markers including relapses and magnetic resonance imaging (MRI) lesion formation in MS (Zimmermann et al., 2018; Lin et al., 2021). However, data on risk stratification and prediction of PIRA remain scarce (Bsteh et al., 2023; Ingwersen et al., 2023).

This study aimed to evaluate OCT‐derived pRNFL and GCIP thicknesses as potential prognostic biomarkers for identifying individuals at risk of sustained PIRA within a heterogenous group of pwRRMS, mimicking real‐world data, and using composite clinical disability measures to comprehensively capture progression across multiple functional domains.

## 2 Methods

### 2.1 Study Design

Participants were retrospectively screened from prospective observational cohort studies conducted at two study centers in Germany: (1) The Berlin cohort at Charité—Universitätsmedizin Berlin, recruited between 2011 and 2021 (CIS‐Cohort [ClinicalTrials.gov: NCT01371071], VIMS) and followed up from 2021 within the BERLimmun study (German Clinical Trial Register: DRKS00026761) (Sperber et al., 2022); (2)The Munich cohort at Technical University of Munich (TUM‐MS), recruited between 2013 and 2021.

The inclusion criteria for this retrospective analysis were (1) a diagnosis of RRMS according to the 2017 revised McDonald criteria (Thompson et al., 2018), (2) age between 18 and 65 years at study inclusion, and (3) at least three visits over a follow‐up period of minimum 2 years. Exclusion criteria were (1) relapses within 3 months prior to baseline and during the follow‐up period, (2) history of bilateral optic neuritis (ON), (3) ophthalmic or systemic comorbidities with potential retinopathy unrelated to MS or potential adverse impact on OCT image acquisition, (4) bilateral refractive errors above ±6 diopters, and (5) no OCT scans acquired at baseline.

**FIGURE 1:**
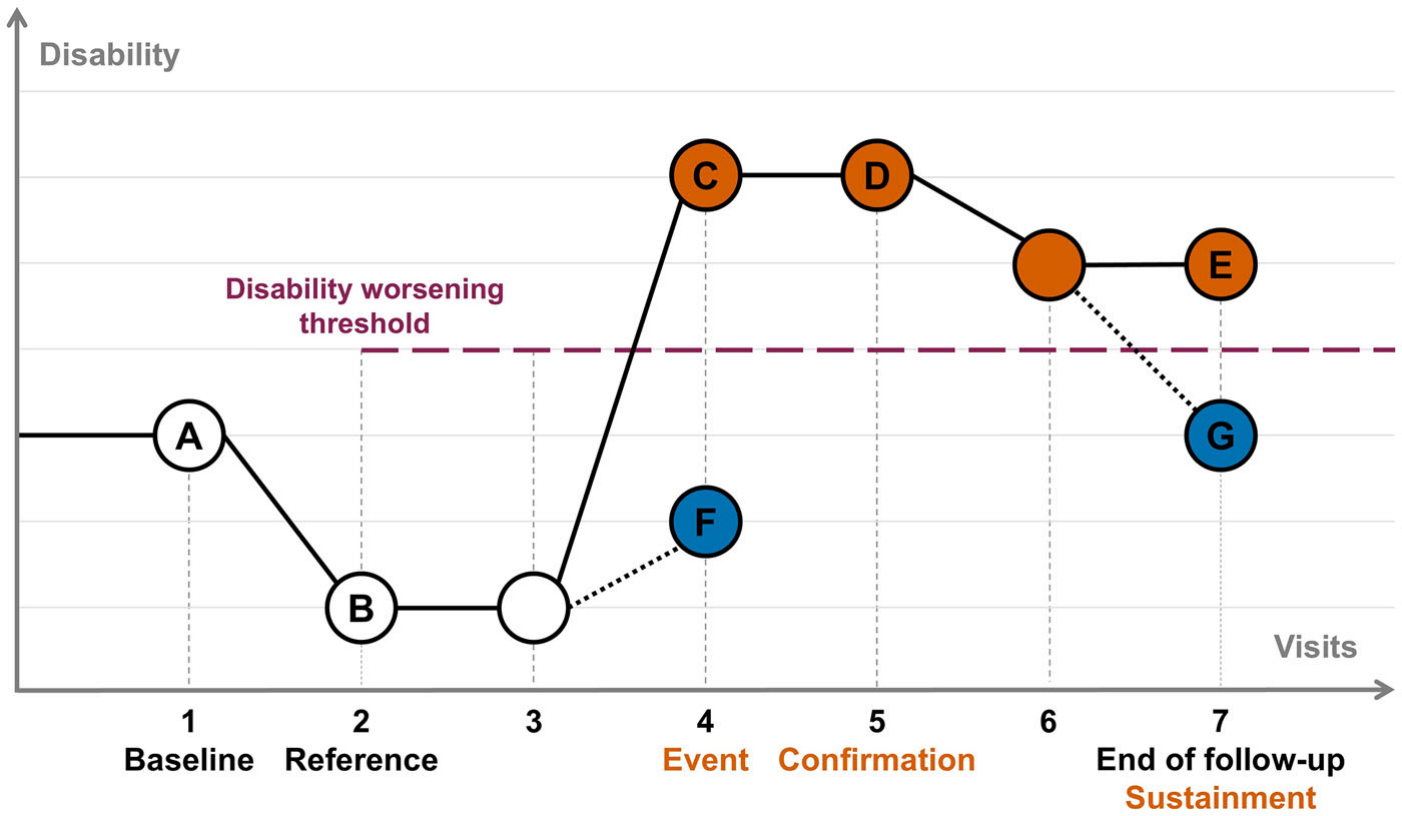
Detection of sustained progression independent of relapse activity (PIRA). Initially, the reference score for each disability measurement is set at baseline (A). However, if the disability improves and is confirmed at the subsequent visit, the reference score is updated to the improved value (B). At this reference, the disability worsening threshold (purple horizontal dashed line) is set for all the following visits. Based on the temporal evolution of disability, two scenarios can occur. Scenario 1 (orange): Sustained PIRA (primary outcome). It is defined as a disability worsening in the absence of relapses above the threshold of clinical significance (C, Event), confirmed at the next consecutive visit (D, Confirmation) and lasting until the end of follow‐up (E, Sustainment). Scenario 2 (blue): Disability worsening below the threshold of clinical significance. This may be the case if the disability worsens but remains below the threshold from the outset (F) or if falls back beneath the threshold during the follow‐up after having previously exceeded it (G). Time between visits ≥ 3 months.

The primary outcome was sustained PIRA. The detection is illustrated in Figure 1 and described in Supporting Information Text S1.

Disability was detected using a composite measurement consisting of the EDSS (≥ 1.5 points increase if reference = 0, ≥ 1.0 points increase if reference 1.0–5.0, ≥ 0.5 points increase if reference ≥ 5.5) (Müller et al., 2023), the T25FW (≥ 20% increase) assessing lower limb function (Meyer‐Moock et al., 2014), the 9HPT (≥ 20% increase) assessing upper limb function (dominant and nondominant hand) (Meyer‐Moock et al., 2014), the SDMT (≥ 4 points or ≥10% decrease) assessing cognitive function (Benedict et al., 2017), and 2.5% low‐contrast letter acuity (LCLA, ≥ 7 letters decrease) assessing visual function (monocular and binocular) (Balcer et al., 2017). A worsening in either one of the measurements can lead to a PIRA event.

### 2.2 Ethics

This study was conducted in accordance with the Declaration of Helsinki in its currently applicable version. All cohort studies were approved by their respective institutional ethics committees at Charité—Universitätsmedizin Berlin (CIS: EA1/182/10, VIMS: EA1/163/12, BERLimmun: EA1/362/20) and the Technical University of Munich (TUM‐MS: 166‐16S/464‐21S/9‐15S). All participants signed a written informed consent prior to study enrolment. We report in adherence to the STROBE (strengthening the reporting of observational studies in epidemiology) Statement.

### 2.3 Optical Coherence Tomography

All OCT scans were quality controlled according to the OSCAR‐IB criteria and are reported based on the APOSTEL 2.0 recommendations (Tewarie et al., 2012; Schippling et al., 2015; Aytulun et al., 2021).

Two distinct scans were analyzed: (1) A peripapillary ring scan centered on the optic nerve head for measuring the pRNFL thickness and (2) a macular volume scan centered on the fovea centralis for calculating the GCIP thickness. Further details regarding the OCT protocol are described in Supporting Information Text S2.

Only eyes without a clinical history of ON were included in the analysis (Petzold et al., 2017). Additionally, to detect subclinical optic nerve involvement, we assessed inter‐eye differences of retinal layer thicknesses with cutoffs of ≥ 6 µm for pRNFL and ≥ 4 µm for GCIP. In case of inter‐eye differences, only the eye with the respective higher values was included (Montalban et al., 2025; Saidha et al., 2025). Furthermore, only eyes without retinopathy unrelated to MS or refractive errors above ±6 diopters were included to ensure independence of other potential confounders. If both eyes of a participant could be included, the mean of both eyes for each retinal layer was calculated and included into analysis.

### 2.4 Age‐adjusted *Z*‐scores

Retinal layer thicknesses are impacted by methodological differences in OCT data acquisition and image post‐processing, as well as by neurodegenerative processes of aging. Therefore, we ensured comparability between study centers and participants by transforming the absolute retinal layer thicknesses of pRNFL and GCIP into age‐adjusted *Z*‐scores as previously described (Lin et al., 2024). Age‐adjusted *Z*‐scores were calculated independently for each study center using generalized additive models for location, scale, and shape, based on center‐specific healthy control cohorts.

### 2.5 Statistical Analysis

The main analysis combined pwRRMS from both study centers into a single cohort. Secondary analyses were then conducted for each study center independently. Exploratory analyses stratified participants by disease duration (≤ 1 year or > 1 year) and sensitivity analyses used different composites of disability measurements (EDSS alone, EDSS or T25FW or 9HPT).

We investigated the association between future risk of sustained PIRA and baseline pRNFL/GCIP age‐adjusted *Z*‐scores by computing univariable and multivariable Cox regression models. The multivariable models were adjusted for age, EDSS, and disease duration (defined as time since the first clinical attack) at baseline, as well as for disease modifying therapy (DMT), treated as a time‐dependent dichotomous variable (yes or no). The selection of covariables was based on reported risk factors for PIRA (Portaccio et al., 2022). We first assessed the adjusted hazard ratios (aHR) and their 95% confidence interval (CI) on a continuous scale, and second, by dichotomizing participants at an age‐adjusted *Z*‐score of −1, −1.5, and −2 into thicker and thinner groups, respectively.

The statistical analysis was performed in R (version 4.4.2). Statistical significance was set as two‐tailed *p* < 0.05. Group comparisons were carried out with Student's *t*‐test, Mann–Whitney *U*‐test or Fisher's exact test as appropriate.

## 3 Results

### 3.1 Baseline Characteristics

**FIGURE 2:**
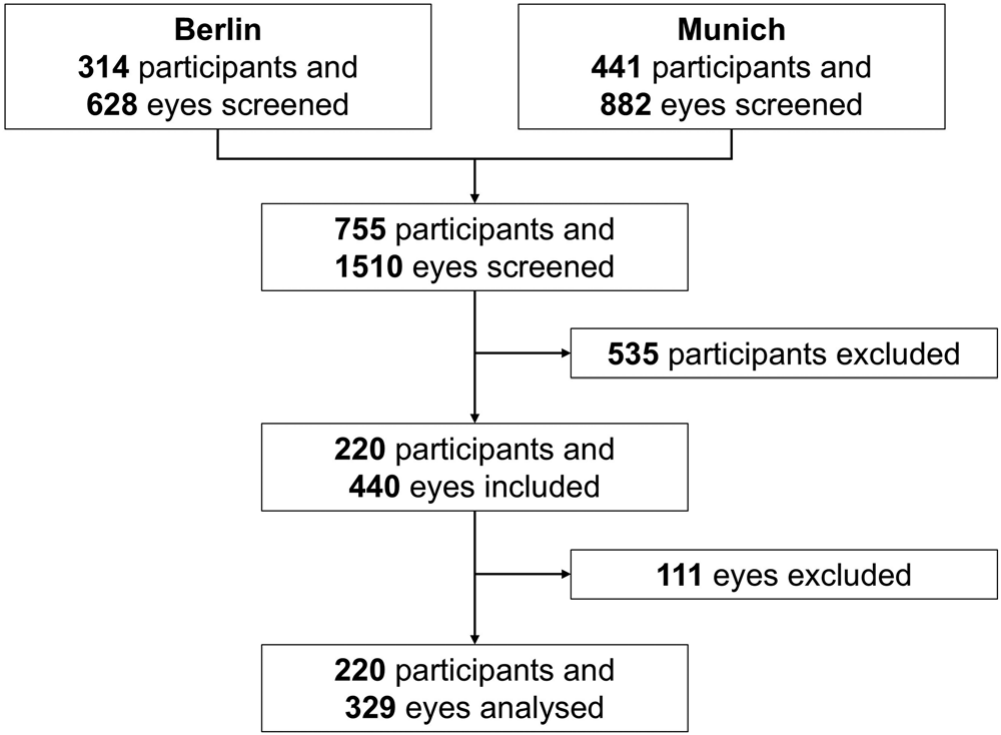
Study centers and numbers of screened, excluded, or included participants and eyes.

As presented in Figure 2, a total of 755 participants were screened, 314 in Berlin and 441 in Munich. Of the screened participants, 220 fulfilled all the inclusion criteria and were included into our analyses, 74 from Berlin and 146 from Munich. Of these included participants, 329 eyes met the inclusion criteria, 115 from Berlin and 214 from Munich.

The baseline characteristics are described and compared between both study centers in Table 1. Information regarding missing data at baseline and during the follow‐up is provided in Supporting Information Table S1.

**TABLE 1:** Baseline characteristics of the study population.

		Study population, *n* = 220	Berlin, *n* = 74	Munich, *n* = 146	*p*, Berlin vs. Munich
**Demographic data**					
Age (years), mean ± SD		38.1 ± 9.9	38.7 ± 10.8	37.8 ± 9.5	0.532
Sex, *n* (%)	Female	130 (59.1)	44 (59.5)	86 (58.9)	1.000
	Male	90 (40.9)	30 (40.5)	60 (41.1)	
Ethnicity, *n* (%)	Caucasian	216 (98.2)	70 (94.6)	146 (100.0)	0.012^*^
	Other	4 (1.8)	4 (5.4)	0 (0)	
**Clinical data**					
Disease duration since first clinical attack (years), median [IQR]		3.7 [0.9–8.3]	1.3 [0.5–9.9]	4.4 [2.2–7.7]	0.034^*^
DMT, *n* (%)	No DMT	56 (25.5)	29 (39.2)	27 (18.5)	< 0.001^*^
	Low/intermediate efficacy	99 (45.0)	34 (45.9)	65 (44.5)	
	High efficacy	65 (29.5)	11 (14.9)	54 (37.0)	
EDSS (score), median [IQR]		1.5 [1.0–2.0]	1.5 [1.0–2.5]	1.0 [0–2.0]	0.051
T25FW (s), median [IQR]		4.2 [3.7–4.7]	4.2 [3.8–4.6]	4.2 [3.6–4.8]	0.934
9HPT dominant hand (s), median [IQR]		17.8 [16.3–20.4]	18.8 [17.3–21.5]	17.2 [16.0–19.5]	< 0.001^*^
9HPT nondominant hand (s), median [IQR]		19.1 [17.4–21.2]	19.8 [17.6–21.8]	18.6 [16.9–21.0]	0.038^*^
SDMT (score), mean ± SD		60.8 ± 13.2	60.2 ± 13.0	66.0 ± 15.3	0.272
Monocular LCLA (letters), median [IQR]		45.0 [40.0–47.3]	45.0 [40.0–47.3]	N/A	N/A
Binocular LCLA (letters), median [IQR]		49.0 [45.0–51.8]	49.0 [45.0–51.8]	N/A	N/A
**Retinal layer thickness**					
pRNFL (µm), mean ± SD		98.7 ± 11.6	96.3 ± 12.2	100.0 ± 11.1	0.026^*^
GCIP (µm), mean ± SD		69.7 ± 6.7	68.5 ± 6.7	70.3 ± 6.6	0.057
pRNFL *Z*‐score, mean ± SD		−0.33 ± 1.45	−0.27 ± 1.45	−0.36 ± 1.45	0.672
GCIP *Z*‐score, mean ± SD		−0.48 ± 1.52	−0.46 ± 1.35	−0.49 ± 1.60	0.906
**Follow‐up**					
Number of visits, median [IQR]		6.0 [5.0–7.0]	5.5 [4.0–7.0]	6.0 [5.0–7.0]	0.755
Follow‐up duration (years), median [IQR]		6.1 [4.0–7.1]	5.4 [3.2–7.4]	6.1 [4.4–7.1]	0.095

*Note*: Retinal layer thickness only of eyes without history of optic neuritis.

Abbreviations: 9HPT = 9‐Hole Peg Test, DMT = disease modifying therapy, EDSS = Expanded Disability Status Scale, GCIP = ganglion cell‐inner plexiform layer, IQR = interquartile range, LCLA = low contrast letter acuity, N/A = not available, pRNFL = peripapillary retinal nerve fiber layer, SD = standard deviation, SDMT = Symbol Digit Modalities Test, T25FW = Timed 25‐Foot Walk Test.

* Statistical significance at *p* < 0.05.

### 3.2 Distribution of sustained PIRA during Follow‐Up

After a median follow‐up duration of 2.9 [IQR 1.6–4.1] years, 65 (29.5%) pwRRMS developed sustained PIRA. Of these, 31 (47.7%) were detected by EDSS increase, 24 (36.9%) by T25FW increase, seven (10.8%) by 9HPT increase, seven (10.8%) by SDMT decrease, and none by LCLA decrease. Sustained PIRA was captured in four (6.2%) pwRRMS by multiple measurements simultaneously.

### 3.3 Association of Baseline pRNFL and GCIP *Z*‐scores with sustained PIRA

**FIGURE 3:**
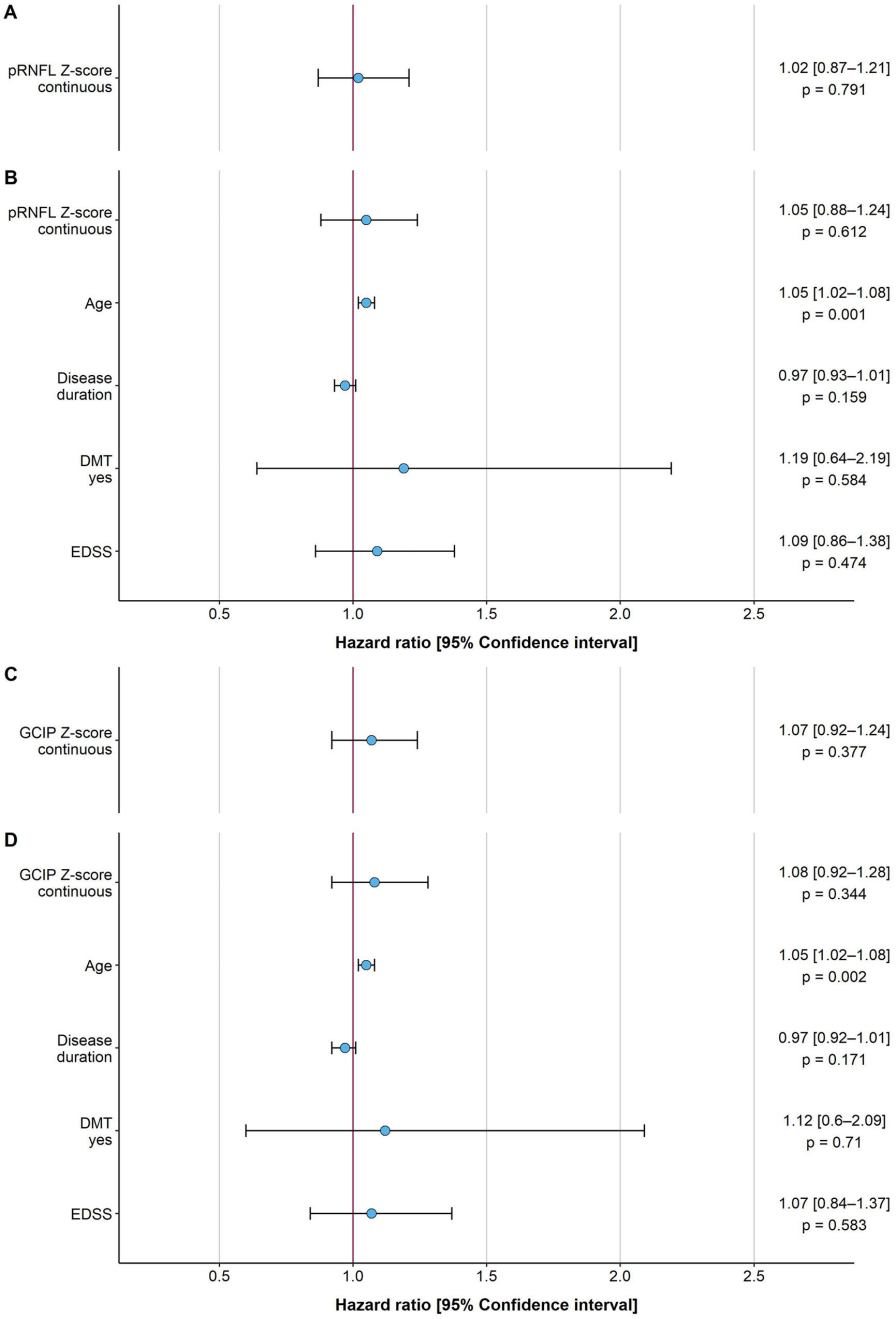
Associations of pRNFL and GCIP age‐adjusted *Z*‐scores on a continuous scale with sustained PIRA for the study population. Univariable (A and C) and multivariable (B and D) Cox proportional hazard models. Dependent variable: Sustained PIRA (progression independent of relapse activity). Independent variable: pRNFL (peripapillary retinal nerve, A and B) or GCIP (ganglion cell‐inner plexiform layer, C and D) as age‐adjusted *Z*‐scores on a continuous scale. Covariables: Age, disease duration since first clinical attack, DMT (disease modifying therapy, dichotomized as yes or no, time‐dependent variable), and EDSS (Expanded Disability Status Scale).

First, we investigated the association of baseline pRNFL and GCIP age‐adjusted *Z*‐scores on a continuous scale with the hazard for sustained PIRA during follow‐up. No associations were found between baseline pRNFL thickness (aHR [95% CI] = 1.05 [0.88–1.24], *p* = 0.612) or GCIP thickness (aHR [95% CI] = 1.08 [0.92–1.28], *p* = 0.344) with sustained PIRA during the follow‐up (Figure 3).

After dichotomizing the participants into two groups at a *Z*‐score cutoff of −2 for each OCT measure, we found no increased risk for sustained PIRA in pwRRMS with thinner pRNFL (aHR [95% CI] = 1.08 [0.52–2.24], *p* = 0.840) or GCIP (aHR [95% CI] = 1.51 [0.78–2.91], *p* = 0.221) at baseline compared to pwRRMS with thicker pRNFL or GCIP (Table 2). Dichotomization at *Z*‐score cutoffs of −1 or −1.5 also showed no risk differences with thinner pRNFL or GCIP respectively but revealed the trend of an increasing aHR for decreasing GCIP *Z*‐score cutoffs (Table 2).

**TABLE 2:** Associations of pRNFL and GCIP age‐adjusted *Z*‐scores below −1, −1.5, and −2 with sustained PIRA for the study population.

Study population (*N* = 220)	*Z* ≤ −1		*Z* ≤ ‐1.5		*Z* ≤ −2	
	aHR [95% CI], *p*‐value					
	*N* = 67		*N* = 48		*N* = 28	
**pRNFL** ^a^	**1.05 [0.60–1.82]**	**0.877**	**0.82 [0.44–1.55]**	**0.546**	**1.08 [0.52–2.24]**	**0.840**
Age^a^	1.05 [1.02–1.08]	0.001^*^	1.05 [1.02–1.08]	0.001^*^	1.05 [1.02–1.08]	0.001^*^
Disease duration^a^	0.97 [0.93–1.01]	0.192	0.98 [0.94–1.02]	0.247	0.97 [0.93–1.01]	0.183
DMT: yes^b^	1.20 [0.65–2.22]	0.565	1.23 [0.66–2.28]	0.508	1.19 [0.64–2.21]	0.574
EDSS^a^	1.09 [0.86–1.38]	0.468	1.09 [0.86–1.38]	0.489	1.09 [0.86–1.39]	0.462
	*N* = 70		*N* = 50		*N* = 29	
**GCIP** ^a^	**0.82 [0.46–1.46]**	**0.504**	**1.15 [0.62–2.13]**	**0.654**	**1.51 [0.78–2.91]**	**0.221**
Age^a^	1.05 [1.02–1.08]	0.002^*^	1.05 [1.02–1.08]	0.003^*^	1.05 [1.02–1.08]	0.003^*^
Disease duration^a^	0.98 [0.93–1.03]	0.373	0.97 [0.85–1.37]	0.230	0.97 [0.93–1.01]	0.177
DMT: yes^b^	1.17 [0.63–2.17]	0.628	1.14 [0.61–2.13]	0.672	1.11 [0.59–2.06]	0.749
EDSS^a^	1.10 [0.86–1.39]	0.454	1.08 [0.85–1.37]	0.535	1.08 [0.85–1.38]	0.516

*Note*: Multivariable Cox proportional hazard models. Dependent variable: Sustained PIRA (progression independent of relapse activity). Independent variable: pRNFL (peripapillary retinal nerve) or GCIP (ganglion cell‐inner plexiform layer) as age‐adjusted *Z*‐scores. Participants dichotomized according to *Z*‐scores into thicker (> −1, −1.5, and −2) and thinner (≤ −1, −1.5, and −2) groups. Covariables: Age, disease duration since first clinical attack, DMT (disease modifying therapy, dichotomized as yes or no), and EDSS (Expanded Disability Status Scale).

Abbreviations: aHR = adjusted hazard ratio, CI = confidence interval.

^a^ Time‐independent variable.

^b^ Time‐dependent variable.

* Statistical significance at *p* < 0.05.

Univariable analyses of *Z*‐score cutoffs are provided in Supporting Information Table S2. Baseline characteristics of pwRRMS with or without sustained PIRA during the follow‐up are compared in Supporting Information Table S3.

### 3.4 Center Differences

We observed clinical differences at baseline between both study centers. In Berlin, more pwRRMS received no DMT (29 [39.2%] vs. 27 [18.5%], *p* < 0.001), had a higher level of disability in 9HPT, a shorter disease duration (1.3 [0.5–9.9] vs. 4.4 [2.2–7.7] years, *p* = 0.034), as well as a numerical higher, though not statistically significant EDSS score (1.5 [1.0–2.5] vs. 1.0 [0–2.0], *p* = 0.051) as compared to the Munich cohort.

After a median follow‐up duration of 2.1 [IQR 1.3–4.0] years, 21 (28.4%) pwRRMS in Berlin experienced an event of sustained PIRA. Of these, seven (33.3%) were detected by EDSS increase, seven (33.3%) by T25FW increase, two (9.5%) by 9HPT increase, and seven (33.3%) by SDMT decrease. After a median follow‐up duration of 3.0 [2.1–4.1] years, 44 (30.1%) pwRRMS in Munich experienced an event of sustained PIRA. Of these, 24 (54.5%) were detected by EDSS increase, 17 (38.6%) by T25FW increase, and five (11.4%) by 9HPT increase.

In Berlin, lower age‐adjusted pRNFL *Z*‐scores on a continuous scale were associated with higher risk for sustained PIRA (aHR [95% CI] = 1.41 [1.03–1.92], *p* = 0.032). A similar association was observed with GCIP, though it did not reach statistical significance in the multivariable analysis (aHR [95% CI] = 1.38 [0.90–2.10], *p* = 0.140). In contrast, pwRRMS in Munich showed no risk differences with either pRNFL or GCIP age‐adjusted *Z*‐scores (Table 3).

**TABLE 3:** Associations of pRNFL and GCIP age‐adjusted *Z*‐scores on a continuous scale with sustained PIRA for Berlin and Munich.

	Univariable		Multivariable	
	HR [95% CI]	*p* value	aHR [95% CI]	*p* value
**Berlin (*n* = 74)**				
**pRNFL** ***Z*‐score continuous** ^a^	**1.49 [1.10–2.02]**	**0.009** ^*^	**1.41 [1.03–1.92]**	**0.032** ^*^
Age^a^			1.03 [0.98–1.09]	0.226
Disease duration^a^			0.95 [0.89–1.02]	0.147
DMT: yes^b^			0.88 [0.35–2.21]	0.782
EDSS^a^			1.56 [0.98–2.48]	0.063
**GCIP *Z*‐score continuous** ^a^	**1.54 [1.06–2.23]**	**0.024** ^*^	**1.38 [0.90–2.10]**	**0.140**
Age^a^			1.02 [0.96–1.08]	0.513
Disease duration^a^			0.96 [0.89–1.04]	0.346
DMT: yes^b^			0.79 [0.30–2.06]	0.630
EDSS^a^			1.47 [0.90–2.41]	0.124
**Munich (*n* = 146)**				
**pRNFL *Z*‐score continuous** ^a^	**0.86 [0.70–1.06]**	**0.151**	**0.90 [0.73–1.11]**	**0.319**
Age^a^			1.05 [1.02–1.09]	0.004^*^
Disease duration^a^			0.98 [0.91–1.05]	0.496
DMT: yes^b^			2.12 [0.63–7.14]	0.224
EDSS^a^			0.94 [0.70–1.27]	0.704
**GCIP *Z*‐score continuous** ^a^	**0.98 [0.82–1.18]**	**0.839**	**1.03 [0.84–1.25]**	**0.780**
Age^a^			1.06 [1.02–1.09]	0.002^*^
Disease duration^a^			0.96 [0.90–1.03]	0.295
DMT: yes^b^			1.67 [0.50–5.60]	0.408
EDSS^a^			0.93 [0.69–1.26]	0.639

*Note*: Univariable and multivariable Cox proportional hazard models. Dependent variable: Sustained PIRA (progression independent of relapse activity). Independent variable: pRNFL (peripapillary retinal nerve) or GCIP (ganglion cell‐inner plexiform layer) as age‐adjusted *Z*‐scores on a continuous scale. Covariables: Age, disease duration since first clinical attack, DMT (disease modifying therapy, dichotomized as yes or no), and EDSS (Expanded Disability Status Scale).

Abbreviations: aHR = adjusted hazard ratio, CI = confidence interval.

^a^ Time‐independent variable.

^b^ Time‐dependent variable.

* Statistical significance at *p* < 0.05.

After dichotomizing the participants at *Z*‐score of −2, pwRRMS in Berlin with thinner pRNFL (aHR [95% CI] = 2.63 [0.82–8.47], *p* = 0.105) or thinner GCIP (aHR [95% CI] = 3.24 [0.95–11.1], *p* = 0.061) had a higher risk for sustained PIRA compared to those above the *Z*‐score cutoff, but both missing statistical significance in the multivariable analysis. Dichotomization at *Z*‐score cutoffs of −1 or −1.5 also showed no significant risk differences with thinner pRNFL or GCIP, respectively but revealed the trend of an increasing aHR for decreasing GCIP Z‐score cutoffs (Table 4). In Munich, we observed no associations between risk of sustained PIRA with either pRNFL or GCIP strata (Supporting Information Table S2).

**TABLE 4:** Associations of pRNFL and GCIP age‐adjusted *Z*‐scores below −1, −1.5, and −2 with sustained PIRA for Berlin.

Berlin (*N* = 74)	*Z* ≤ −1		*Z* ≤ ‐1.5		*Z* ≤ −2	
	aHR [95% CI], *p*‐value					
	*N* = 24		*N* = 16		*N* = 6	
**pRNFL** ^a^	**2.39 [0.89–6.44]**	**0.084**	**1.34 [0.47–3.84]**	**0.589**	**2.63 [0.82–8.47]**	**0.105**
Age^a^	1.03 [0.89–1.09]	0.231	1.04 [0.98–1.10]	0.201	1.04 [0.99–1.09]	0.145
Disease duration^a^	0.95 [0.88–1.02]	0.161	0.96 [0.90–1.03]	0.282	0.96 [0.90–1.03]	0.251
DMT: yes^b^	0.84 [0.34–2.08]	0.711	0.79 [0.32–1.98]	0.617	0.85 [0.33–2.15]	0.724
EDSS^a^	1.62 [1.01–2.60]	0.043*	1.63 [1.02–2.61]	0.042*	1.52 [0.94–2.44]	0.085
	*N* = 27		*N* = 17		*N* = 8	
**GCIP** ^a^	**1.06 [0.37–3.01]**	**0.913**	**1.64 [0.55–4.91]**	**0.373**	**3.24 [0.95–11.1]**	**0.061**
Age^a^	1.03 [0.97–1.09]	0.415	1.02 [0.97–1.08]	0.406	1.01 [0.96–1.07]	0.690
Disease duration^a^	0.97 [0.90–1.05]	0.492	0.97 [0.89–1.05]	0.397	0.97 [0.89–1.04]	0.387
DMT: yes^b^	0.75 [0.29–1.95]	0.554	0.79 [0.30–2.06]	0.626	0.80 [0.31–2.11]	0.657
EDSS^a^	1.60 [0.98–2.59]	0.059	1.54 [0.94–2.51]	0.086	1.57 [0.97–2.55]	0.065

*Note*: Multivariable Cox proportional hazard models. Dependent variable: Sustained PIRA (progression independent of relapse activity). Independent variable: pRNFL (peripapillary retinal nerve) or GCIP (ganglion cell‐inner plexiform layer) as age‐adjusted *Z*‐scores. Participants dichotomized according to *Z*‐scores into thicker (> −1, −1.5, and −2) and thinner (≤ −1, −1.5, and −2) groups. Covariables: Age, disease duration since first clinical attack, DMT (disease modifying therapy, dichotomized as yes or no), and EDSS (Expanded Disability Status Scale).

Abbreviations: aHR = adjusted hazard ratio, CI = confidence interval.

^a^ Time‐independent variable.

^b^ Time‐dependent variable.

* Statistical significance at *p* < 0.05.

**FIGURE 4:**
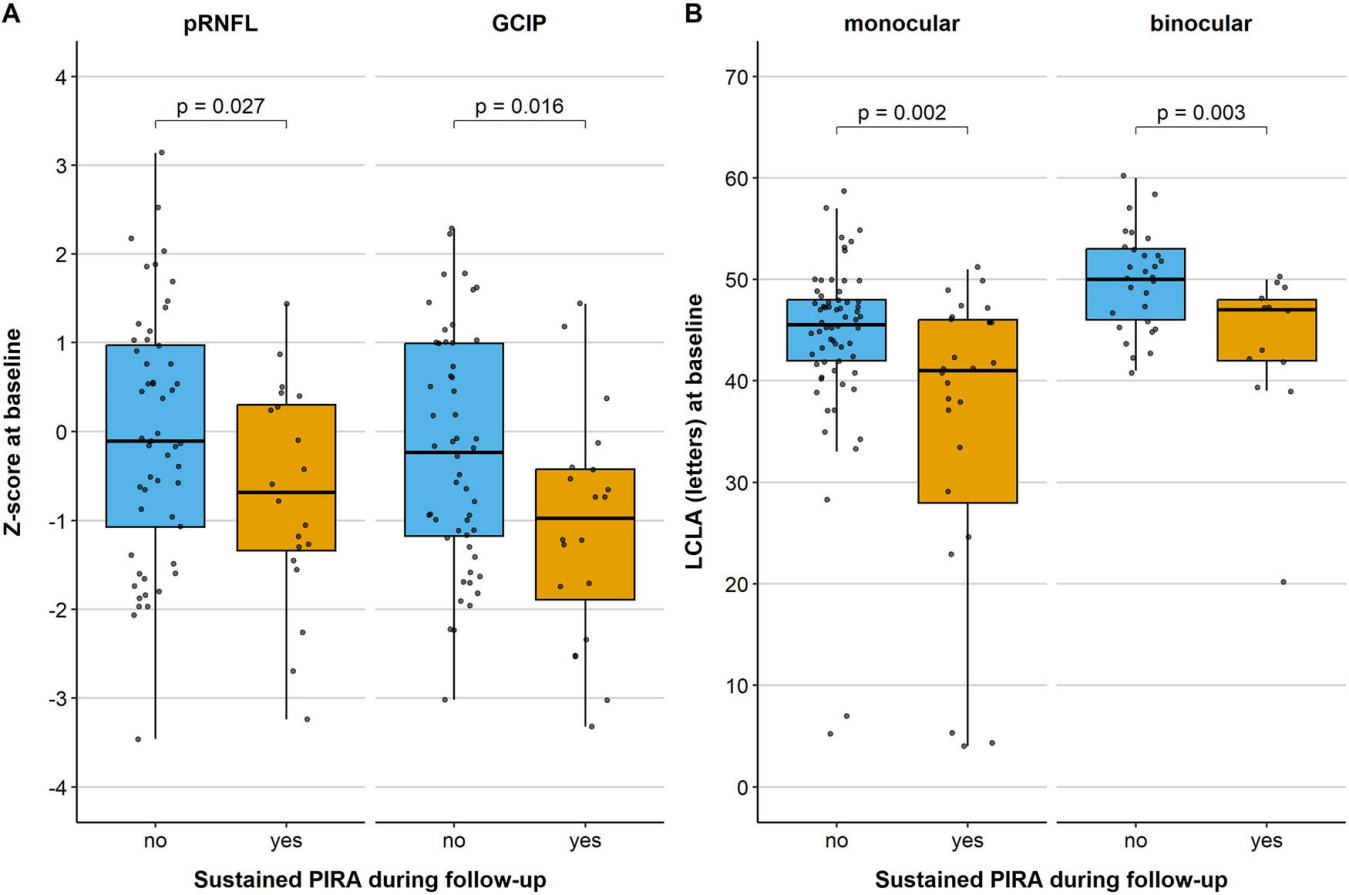
Boxplots of baseline differences in visual measures between people with relapsing‐remitting multiple sclerosis in Berlin who developed sustained progression independent of relapse activity (PIRA) during the follow‐up (orange) compared to those who did not (blue). (A) Thicknesses of the peripapillary retinal nerve fiber layer (pRNFL; as *Z*‐scores: −0.86 ± 1.44 vs. −0.04 ± 1.40, *p* = 0.027; in µm: 91.0 ± 10.8 vs. 98.4 ± 12.3 µm, *p* = 0.019) and ganglion cell‐inner plexiform layer (GCIP; as *Z*‐scores: −1.08 ± 1.29 vs. −0.23 ± 1.30, *p* = 0.016; in µm: 65.2 ± 6.5 vs. 69.7 ± 6.4 µm, *p* = 0.010). (B) Low‐contrast letter acuity (LCLA) monocular (41.0 [28.0–46.0] vs. 45.5 [42.0–48.0] letters, *p* = 0.002) and binocular (47.0 [42.0–48.0] vs. 50.0 [46.0–53.0] letters, *p* = 0.003).

In Berlin, pwRRMS who developed sustained PIRA during follow‐up had lower baseline pRNFL and GCIP thicknesses and performed worse on monocular and binocular LCLA compared to those without sustained PIRA (Figure 4). In Munich, pwRRMS with sustained PIRA during the follow‐up were older at baseline as compared to those without sustained PIRA (40.9 ± 8.9 vs. 36.5 ± 9.4 years, *p* = 0.008).

### 3.5 Differences between earlier and later Disease Stages

After dividing pwRRMS by disease duration across both study centers, we observed that pwRRMS within the first year after disease onset (*n* = 57 [Berlin 63.2%, Munich 36.8%]) did not have an increased risk for sustained PIRA with decreasing age‐adjusted *Z*‐scores of pRNFL (aHR [95% CI] = 1.28 [0.88–1.88], *p* = 0.202) or GCIP (aHR [95% CI] = 1.16 [0.77–1.73], *p* = 0.474) (Supporting Information Table S4). Similarly, we found no associations between pRNFL or GCIP thickness and sustained PIRA in pwRRMS at a later disease stage (> 1 year, *n* = 162 [Berlin 22.8%, Munich 77.2%]).

### 3.6 Defining sustained PIRA by other composites of Disability Measurements

We observed substantial differences in missing data for LCLA and SDMT between Berlin and Munich (Supporting Information Table S1). After defining sustained PIRA solely by worsening in EDSS, T25FW, and 9HPT, which all had similar proportions of missing data across both study centers, decreasing age‐adjusted *Z*‐scores of pRNFL (aHR [95% CI] = 1.02 [0.85–1.22], *p* = 0.834) or GCIP (aHR [95% CI] = 1.07 [0.90–1.27], *p* = 0.439) remained not associated with sustained PIRA within the overall study population (Supporting Information Table S5).

## 4 Discussion

We retrospectively analyzed OCT‐derived age‐adjusted pRNFL and GCIP *Z*‐scores as predictive biomarkers for sustained PIRA in pwRRMS, using data from prospective observational cohorts conducted at two German study centers. No associations were found between baseline pRNFL or GCIP thickness and sustained PIRA within the overall study population. However, subgroup analyses revealed contradictory results. In the Berlin cohort, characterized by shorter disease durations and higher disability levels compared to the Munich cohort, pwRRMS with thinner pRNFL or GCIP had an increased risk for sustained PIRA compared to those with thicker measures. In contrast, no such associations were found within the Munich cohort.

Only a few studies have previously analyzed associations between OCT‐derived measures and PIRA (Bsteh et al., 2020, 2023; Ingwersen et al., 2023; Burguet Villena et al., 2025). Two aimed to predict the hazard of PIRA with baseline pRNFL and GCIP thickness. (Bsteh et al., 2023; Ingwersen et al., 2023) These studies had contradictory results that parallel our findings: The first study analyzed 231 pwRRMS shortly after diagnosis (mean age: 30.3 years, median disease duration: 45 days, females: 74%, moderate efficacy DMT: 59.7%, high efficacy DMT: 27.3%, median EDSS: 0, mean pRNFL: 91.9 µm, mean GCIP: 80.2 µm) and showed that thinner pRNFL and GCIP predicted PIRA (Bsteh et al., 2023). The second study analyzed 97 pwRRMS at later disease stages (mean age: 42.3 years, mean disease duration: 10.5 years, females: 48.6%, DMT Ocrelizumab: 100%, mean EDSS: 3.5, mean pRNFL: 81 µm, mean GCIP: 59 µm [1.68 mm^3^]) and found no associations between pRNFL or GCIP and PIRA (Ingwersen et al., 2023).

These findings suggest a potential prognostic value of pRNFL and GCIP thickness for predicting PIRA. The clinical differences hint that they are particularly promising in earlier, but not later disease stages. However, exploratory analyses stratifying our entire study population by disease duration (below or above 1 year) did not confirm this, questioning the validity of our center‐specific observation. Since pRNFL and GCIP reflect neuroaxonal damage, our findings might add to the emerging evidence of neurodegenerative processes occurring from disease onset and emphasize the beginning of the disease course as a vulnerable phase for the long‐term outcome, which current treatment options might not properly address. These mechanisms of progression include chronic active inflammation, chronic demyelination, as well as metabolic and mitochondrial dysfunction (Kuhlmann et al., 2023). Because retinal layer thickness and atrophy reflect a common final pathway of neuroaxonal damage, OCT‐derived measures cannot distinguish between the underlying pathological drivers and will be required to be combined with other biomarkers to properly capture the underlying disease processes (Lin and Saidha, 2025).

Besides clinical heterogeneity, further aspects limit the comparability of previous studies (Bsteh et al., 2023; Ingwersen et al., 2023) with each other and with our findings, which should be taken into consideration when interpreting the evidence. Most importantly, the definition of PIRA varies considerably across studies. The rapid emergence of this concept in recent years has sparked an ongoing debate regarding the definition as multiple studies have shown that the definition used impacts the incidence and persistence of PIRA events (Kappos et al., 2018, 2020; Müller et al., 2025; Satyanarayan et al., 2023; Sharrad et al., 2023). This issue has recently been addressed (Müller et al., 2023, 2025). The two previous studies defined PIRA solely by EDSS worsening confirmed after 3 or 6 months respectively, using a fixed baseline and allowing relapses except within certain intervals (Bsteh et al., 2023; Ingwersen et al., 2023). In contrast, a major strength of our study is the alignment of PIRA with core pillars of recently proposed harmonizing definitions (Müller et al., 2023, 2025). We increased sensitivity for detecting PIRA by examining clinical measurements beyond the EDSS to account for multiple dimensions of disability within a composite, and applying a roving baseline to adjust for improvements in disability (Kappos et al., 2018, 2020; Müller et al., 2023). To avoid overestimation of PIRA, we also increased specificity by requiring PIRA to be sustained until the end of follow‐up to capture clinically meaningful long‐term disability worsening, and excluding pwRRMS with relapses during the follow‐up to mitigate the influence of clinical disease activity (Lublin et al., 2022; Satyanarayan et al., 2023). However, a critical limitation shared by both previous studies and ours is the absence of MRI analyses to assess subclinical disease activity. (Bsteh et al., 2023; Ingwersen et al., 2023). Recent studies have shown, that up to half of pwRRMS with PIRA, especially those under low‐efficacy DMTs, continue to show focal inflammatory MRI activity in temporal relation to a PIRA event (Tur et al., 2023; Strijbis et al., 2025). This challenges whether the pure clinical definition of PIRA truly reflects an underlying neurodegeneration independent of focal inflammation (Ciccarelli et al., 2024). This emphasizes the importance of incorporating MRI to account for different pathological disease processes within MS and PIRA, leading to the refined concept of *progression independent of relapse and MRI activity* (PIRMA), which may better isolate true progression outside the bounds of focal inflammation (Lin and Saidha, 2025; Calabrese et al., 2024).

Another key aspect concerns the OCT methodology and retinal layer thickness measures. Both previously mentioned studies used spectral domain OCT (Spectralis OCT1 or OCT2) with Heidelberg Eye Explorer (both by Heidelberg Engineering, Heidelberg, Germany) for image acquisition and postprocessing (Bsteh et al., 2023; Ingwersen et al., 2023). We used the same devices and software in our study, with the exception of the SAMIRIX pipeline for segmenting GCIP in the Berlin cohort (Motamedi et al., 2019). Despite that, we see large differences in the pRNFL and GCIP thicknesses across all studies and cohorts, only to certain extent explainable by different OCT software versions and post‐processing pipelines or different clinical characteristics mentioned beforehand. We tried to address this issue by transforming absolute thicknesses of pRNFL and GCIP into age‐adjusted *Z*‐scores, which have been shown to outperform absolute values as predictors and improve the comparability between different OCT devices (Lin et al., 2024). With this, we equalized center differences regarding OCT methodology and improved comparability between both cohorts for pRNFL and GCIP (Berlin vs. Munich; Table 1).

Our study has several crucial limitations. First, and most importantly, we did not detect subclinical inflammatory disease activity through MRI to ensure PIRMA (Strijbis et al., 2025; Ciccarelli et al., 2024). Due to inconsistent MRI protocols within the follow‐up period, we refrained ourselves from including them into the analyses. As such, mild or unspecific symptoms may have gone unrecognized, potentially leading to inclusion of participants with subtle inflammatory disease activity. Furthermore, the exclusion of pwRRMS with relapses raises the question of whether the occurrence of sustained PIRA marks a transition to secondary‐progressive MS (Portaccio et al., 2024). Second, despite our multidimensional approach to disability, the measurements used only assess fractions of certain disability domains. For example, SDMT detected only half of cognitive PIRA events in a recent study (Fuchs et al., 2024). Nevertheless, a balance between comprehensive testing and clinical practicability must be maintained. Additionally, we observed substantial differences in missing data for LCLA and SDMT between Berlin and Munich. Therefore, we conducted sensitivity analyses defining sustained PIRA solely by worsening in EDSS, T25FW, or 9HPT, which all had similar proportions of missing data, and observed consistent results with our main analyses. Third, we did not analyze the effects of distinct DMTs or treatment hierarchies, although they influence the occurrence of PIRA (Müller et al., 2023). However, we adjusted for treatment duration by including DMT (yes/no) as a time‐dependent variable into our models and found no associations with sustained PIRA. Last, our study population predominantly consists of pwRRMS with Caucasian ethnicity, which limits generalizability. Furthermore, the small number of pwRRMS with disease duration < 1 year impairs exploratory analyses on the utility of pRNFL and GCIP as biomarkers for PIRA in early MS. Therefore, the inconsistent findings across centers may also be attributable to sample noise rather than true biological differences.

Future studies should address these limitations through larger, prospective and multicentric designs with the inclusion of MRI to improve our understanding of PIRA/PIRMA and the utility of pRNFL and GCIP thickness as biomarkers for risk stratification. Additionally, age‐matched healthy controls should be incorporated, since age is the single risk factor for PIRA in pwRRMS that remains consistent across most studies (Portaccio et al., 2022; Tur et al., 2023; Müller et al., 2025; Prosperini et al., 2024), and was also seen in ours. Including age‐matched controls would further advance our understanding of the relationships among MS, PIRA/PIRMA, and age. Furthermore, other OCT‐based methods such as OCT‐Angiography might harbor potential to be investigated in the future.

In conclusion, OCT‐derived pRNFL and GCIP measures may serve as prognostic imaging biomarkers to identify pwRRMS at risk of PIRA. While they do not predict the hazard of PIRA within a heterogenous group of pwRRMS, they appear to be particularly promising in early disease stages. Further research is required to establish a foundation for the application of OCT measures, focusing on the influence of clinical characteristics, to find out which patient groups could benefit from routine assessments to reliably predict PIRA/PIRMA.

## Author Contributions

**Andre Braginets**: conceptualization, data curation, formal analysis, investigation, methodology, visualization, writing – original draft preparation. **Frederike Cosima Oertel**: data curation, investigation, writing – review and editing. **Eva‐Maria Strauß**: data curation, investigation, writing – review and editing. **Carla Leutloff**: Data curation, investigation, writing – review and editing. **Tanja Schmitz‐Hübsch**: investigation, resources, writing – review and editing. **Friedemann Paul**: funding acquisition, resources, writing – review and editing. **Susanna Asseyer**: resources, writing – review and editing. **Rebecca Wicklein**: writing – review and editing. **Bernhard Hemmer**: funding acquisition, resources, writing – review and editing. **Klemens Ruprecht**: resources, writing – review and editing. **Hanna Gwendolyn Zimmermann**: conceptualization, formal analysis, project administration, supervision, writing – review and editing. **Benjamin Knier**: conceptualization, funding acquisition, resources, supervision, writing – review and editing. **Ting‐Yi Lin**: conceptualization, data curation, formal analysis, methodology, supervision, writing – review and editing

## Funding statement

This study was supported by a research grant from Novartis Pharma GmbH (to Friedemann Paul). Friedemann Paul was additionally funded by the Deutsche Forschungsgemeinschaft (DFG, German Research Foundation) under Germany's Excellence Strategy—EXC‐2049–390688087. Benjamin Knier was funded by the DFG under 528297171. Bernhard Hemmer was supported by the European Union's Horizon 2020 Research and Innovation Program [WISDOM EU RIA 101137154], the Deutsche Forschungsgemeinschaft (DFG, German Research Foundation) under Germany's Excellence Strategy within the framework of the Munich Cluster for Systems Neurology [EXC 2145 SyNergy—ID 390857198], and the Clinspect‐M consortium funded by the Bundesministerium für Bildung, Wissenschaft, Forschung und Technologie.

## Ethics Statement

All cohort studies were approved by their respective institutional ethics committees at Charité—Universitätsmedizin Berlin (CIS: EA1/182/10 at 9th of September 2010, VIMS: EA1/163/12 at 21st of August 2012, BERLimmun: EA1/362/20 at 8th of July 2021) and the Technical University of Munich (TUM‐MS: 166‐16S/464‐21S/9‐15S at 31st of March 2016/23rd of June 2021/22nd of January 2015 respectively).

## Consent

All participants signed a written informed consent prior to study enrolment.

## Conflicts of Interest

The author(s) declared the following potential conflicts of interest with respect to the research, authorship, and/or publication of this article: Andre Braginets, Eva‐Maria Strauß, Carla Leutloff, Rebecca Wicklein and Ting‐Yi Lin report no relevant disclosures. Frederike Cosima Oertel reports current research grants by DFG (TRR418), Hertie foundation, and Novartis—not related to this project; past fellowship support by the American Academy of Neurology and the National MS Society; past research grant by the DGN (Germany Neurology Association) and DFG‐TWAS program; speaker honoraria by UCB and Novartis; travel support by Guthy Jackson Charitable Foundation, European Committee for Research and Treatment in Multiple Sclerosis, and American Academy of Neurology; academic editor at DGNeurologie and Neurological Research & Practice; Board member at the IMSVISUAL consortium. Tanja Schmitz‐Hübsch received research grants from Celgene/bms and speakers honoraria from Roche. Friedemann Paul received research support from Bayer, Novartis, Biogen, Teva, Sanofi‐Aventis/Geynzme, Alexion, and Merck Serono; the German Research Council; Werth Stiftung of the City of Cologne; German Ministry of Education and Research; Arthur Arnstein Stiftung Berlin; EU FP7 Framework Program; Arthur Arnstein Foundation Berlin; Guthy‐Jackson Charitable Foundation; and NMSS. He also reports consulting fees as associate editor of Neurology; Neuroimmunology & Neuroinflammation as academic editor of PLoS ONE; and as consultant for Sanofi Genzyme, Biogen, MedImmune, Shire, and Alexion. He also received speaker honoraria from Bayer, Novartis, Biogen, Teva, Sanofi‐Aventis/Genzyme, Merck Serono, Alexion, Chugai, MedImmune, and Shire and travel grants from Bayer, Novartis, Biogen, Teva, Sanofi‐Aventis/Genzyme, Merck Serono, Alexion, Chugai, MedImmune, and Shire. Susanna Asseyer received speaker's honoraria from Bayer, Alexion, Roche, and research grants from Stiftung Charité, Fritz‐Thyssen‐Stiftung, HEAD Genuit Stiftung, Rahel Hirsch Program, Novartis, and Roche, unrelated to this project. Bernhard Hemmer served on scientific advisory boards for Novartis, Hoffmann LaRoche, Amgen, and Polpharma; he has served as DMSC member for AllergyCare, Sandoz, Polpharma, Biocon, and TG therapeutics; his institution received research grants from Hoffmann LaRoche and Polpharma for multiple sclerosis research. He provided a declaration statement on DMT for the law firm Wuesthoff & Wuesthoff. He has received honoraria for counseling (Gerson Lehrmann Group, AlphaSights Ltd). Klemens Ruprecht received research support from Novartis, Merck Serono, German Ministry of Education and Research, European Union (821283‐2), Stiftung Charité, Guthy‐Jackson Charitable Foundation, and Arthur Arnstein Foundation; travel grants from Guthy‐Jackson Charitable Foundation; speaker's honoraria from Virion Serion and Novartis; and was a participant in the BIH Clinical Fellow Program funded by Stiftung Charité. Hanna Gwendolyn Zimmermann received research grants and speaking honoraria from Novartis. Benjamin Knier received travel support and speaking honoraria from Alexion, Argenx, Heidelberg Engineering, Hexal, Merck, Novo Nordisk, Novartis, Teva, and Roche and served at the advisory boards of Alexion and Merck.

## Supporting information




**Supplementary Materials**: brb371612‐sup‐0001‐SuppMat.docx

## Data Availability

Written requests for access to the data reported in this paper will be considered by the corresponding author and a decision made about the appropriateness of the use of the data. If deemed appropriate, a data‐sharing agreement will be established prior to providing access to a fully de‐identified version of the dataset.
