# Supplementary material for: Retinal Layer Thickness for Risk Stratification of Progression Independent of Relapse Activity in Relapsing‐Remitting Multiple Sclerosis
Source: Brain Behav. 2026 Jul 28;16(8):e71612. doi: 10.1002/brb3.71612 (PMC13411297; doi:10.1002/brb3.71612)
Supplement: Supplementary file 1 — Supplementary Materials: brb371612‐sup‐0001‐SuppMat.docx [file BRB3-16-e71612-s001.docx]

**Supplement**

[Supplementary Text S1. Detection of PIRA 2](#_Toc231405541)

[Supplementary Text S2. OCT Protocol 3](#_Toc231405542)

[Supplementary Table S1. Missing data 4](#_Toc231405543)

[Supplementary Table S2. Z-score cutoffs 5](#_Toc231405544)

[S2a. Univariable analyses: Study population, Berlin and Munich 5](#_Toc231405545)

[S2b. Multivariable analyses: Munich 7](#_Toc231405546)

[Supplementary Table S3. PwRRMS without vs with sustained PIRA 8](#_Toc231405547)

[Supplementary Table S4. Exploratory analyses stratified by disease duration 10](#_Toc231405548)

[Supplementary Table S5. Sensitivity analyses by different disability composites 12](#_Toc231405549)

[S5a. EDSS 12](#_Toc231405550)

[S5b. EDSS-Plus (EDSS or T25FW or 9HPT) 13](#_Toc231405551)

# **Supplementary Text S1. Detection of PIRA**

Study visits were conducted annually if possible. Only follow-ups that took place ≥3 months after the previous visit were included in the analyses.

The visit, which sets the reference score for each disability measurement assed and the threshold, to which subsequent visits are compared to for detecting a significant increase of disability, is called the *reference*. It is the baseline visit at study-onset, but it can row during the follow-up and be “re-baselined” if either the disability score improves and is being confirmed at the next consecutive visit or if a confirmed PIRA event occurs. There may be different reference visits for each disability measurement.

The visit, where a significant increase of disability in either one of the disability measurements is detected, is referred to as the *event*.

For the PIRA event to be confirmed, the disability score must remain above the threshold of the significant increase of disability until the next consecutive visit, which is referred to as the *confirmation*.

For the PIRA event to be sustained, the disability score must remain above the threshold of the significant increase of disability (which was set at the reference visit of the PIRA event) until the end of follow-up, which is referred to as the *sustainment*. If possible, the confirmation and sustainment are distinct visits, but in case of the confirmation being the last follow-up visit, it can be both simultaneously, if the confirmation visit is more than 12 months after the event.

# **Supplementary Text S2. OCT Protocol**

The OCT examinations were performed under normal room light by experienced operators and on the same day as the disability measurements if possible. The pupils of the participants were not pharmacologically dilated before examination.

In Berlin, the scans were acquired by spectral domain OCT (prior to 2018 with Spectralis OCT1, from 2018 onwards with Spectralis OCT2) with Heidelberg Eye Explorer (HeyEx) version 1.9.10.0 (both by Heidelberg Engineering, Heidelberg, Germany). Peripapillary ring scans were segmented with HeyEx viewing module version 6.0.14.0. Macular volume scans were segmented with the semiautomated SAMIRIX pipeline.

In Munich, the scans were acquired by spectral domain OCT (Spectralis OCT2) with HeyEx version 2.5.4 (both by Heidelberg Engineering, Heidelberg, Germany). Both, peripapillary ring scans and macular volume scans were segmented with HeyEx viewing module version 6.9.a.

In both centers segmentation errors were manually assessed and corrected if necessary.

Two distinct scans were analyzed: (1) A peripapillary ring scan (1536 A-scans, automatic real-time tracking [ART]: 9 - 99) with a 12° (3.4 or 3.5 mm) diameter centered on the optic nerve head for measuring the pRNFL thickness. (2) A macular volume scan (25° x 30°, 61 vertical B-scans, ART: 12 - 18) for calculating the GCIP thickness with an extracted 6 mm diameter cylinder centered on the fovea centralis (Early Treatment Diabetic Retinopathy Study grid). The thicknesses were defined for both scans as the global mean of all retinal sectors.

# **Supplementary Table S1. Missing data**

|  | Missing at baseline, n (%) | | Incomplete follow-up, n (%) | |
| --- | --- | --- | --- | --- |
|  | **Berlin (n=74)** | **Munich (n=146)** | **Berlin (n=74)** | **Munich (n=146)** |
| Disease Duration | 1 (1.4) | 0 (0) | - | - |
| EDSS | 0 (0) | 0 (0) | 5 (6.8) | 0 (0) |
| T25FW | 3 (4.1) | 10 (6.8) | 22 (29.7) | 30 (20.5) |
| 9HPT | 3 (4.1) | 10 (6.8) | 20 (27.0) | 30 (20.5) |
| SDMT | 4 (5.4) | 139 (95.2) | 24 (32.4) | 132 (90.4) |
| LCLA monocular | 28 (37.8) | N/A | 42 (56.8) | N/A |
| LCLA binocular | 32 (43.2) | N/A | 57 (77.0) | N/A |
| pRNFL | 0 (0) | 3 (2.1) | - | - |
| GCIP | 2 (2.7) | 2 (1.4) | - | - |

*Missing data at baseline or during the follow-up. Incomplete follow-up is defined as missing data in at least one follow-up visit. In case of missing values, no imputations were conducted. EDSS = Expanded Disability Status Scale, T25FW = Timed 25-Foot Walk Test, 9HPT = 9-Hole Peg Test, SDMT = Symbol Digit Modalities Test, LCLA = Low Contrast Letter Acuity, pRNFL = peripapillary retinal nerve fiber layer, GCIP = combined ganglion cell-inner plexiform layer. N/A = not available.*

# **Supplementary Table S2. Z-score cutoffs**

# **S2a. Univariable analyses: Study population, Berlin and Munich**

| Study population  (N = 220) | Z ≤ -1 | | Z ≤ 1.5 | | Z ≤ -2 | |
| --- | --- | --- | --- | --- | --- | --- |
|  | aHR [95% CI], p-value | | | | | |
|  | N = 67 | | N = 48 | | N = 28 | |
| pRNFL | 1.09  [0.65 - 1.85] | 0.736 | 0.86  [0.47 - 1.58] | 0.626 | 1.00  [0.49 - 2.02] | 0.996 |
|  | N = 70 | | N = 50 | | N = 29 | |
| GCIP | 0.95  [0.56 - 1.61] | 0.838 | 1.33  [0.75 - 2.35] | 0.332 | 1.63  [0.87 - 3.06] | 0.129 |

| Berlin  (N = 74) | Z ≤ -1 | | Z ≤ 1.5 | | Z ≤ -2 | |
| --- | --- | --- | --- | --- | --- | --- |
|  | aHR [95% CI], p-value | | | | | |
|  | N = 24 | | N = 16 | | N = 6 | |
| pRNFL | 2.63  [1.09 - 6.39] | 0.032* | 1.65  [0.59 - 4.60] | 0.341 | 3.78  [1.25 - 11.4] | 0.018* |
|  | N = 27 | | N = 17 | | N = 8 | |
| GCIP | 1.63  [0.67 - 3.95] | 0.280 | 2.39  [0.92 - 6.18] | 0.072 | 4.15  [1.46 - 11.8] | 0.007* |

| Munich  (N = 146) | Z ≤ -1 | | Z ≤ 1.5 | | Z ≤ -2 | |
| --- | --- | --- | --- | --- | --- | --- |
|  | aHR [95% CI], p-value | | | | | |
|  | N = 43 | | N = 32 | | N = 22 | |
| pRNFL | 0.68  [0.34 – 1.35] | 0.272 | 0.63  [0.29 - 1.35] | 0.234 | 0.58  [0.23 - 1.48] | 0.256 |
|  | N = 43 | | N = 33 | | N = 21 | |
| GCIP | 0.70  [0.35 - 1.43] | 0.328 | 0.99  [0.48 - 2.08] | 0.988 | 1.09  [0.49 - 2.46] | 0.827 |

*Associations of pRNFL and GCIP age-adjusted Z-scores below -1, -1.5 and -2 with sustained PIRA for the study population, Berlin and Munich. Univariable Cox proportional hazard models. Dependent variable: Sustained PIRA (progression independent of relapse activity). Independent variable: pRNFL (peripapillary retinal nerve) or GCIP (GCIP = ganglion cell-inner plexiform layer) as age-adjusted Z-scores. Participants dichotomized according to Z-scores into thicker (> -1, -1.5 and -2) and thinner (≤ -1, -1.5 and -2) groups. aHR = adjusted hazard ratio, CI = confidence interval. * Statistical significance p < 0.05*

# **S2b. Multivariable analyses: Munich**

| Munich  (N = 146) | Z ≤ -1 | | Z ≤ 1.5 | | Z ≤ -2 | |
| --- | --- | --- | --- | --- | --- | --- |
|  | aHR [95% CI], p-value | | | | | |
|  | N = 43 | | N = 32 | | N = 22 | |
| pRNFL^†^ | **0.66**  **[0.32 - 1.36]** | **0.260** | **0.60**  **[0.27 - 1.35]** | **0.214** | **0.59**  **[0.22 - 1.56]** | **0.285** |
| Age^†^ | 1.05  [1.02 - 1.09] | 0.002* | 1.05  [1.02 - 1.09] | 0.002* | 1.05  [1.02 - 1.09] | 0.003* |
| Disease duration^†^ | 0.98  [0.91 - 1.05] | 0.520 | 0.98  [0.91 - 1.05] | 0.552 | 0.98  [0.91 - 1.05] | 0.512 |
| DMT: yes^‡^ | 2.17  [0.64 – 7.35] | 0.213 | 2.19  [0.65 - 7.39] | 0.207 | 2.19  [0.65 - 7.36] | 0.206 |
| EDSS^†^ | 0.95  [0.70 - 1.27] | 0.714 | 0.94  [0.70 - 1.26] | 0.659 | 0.93  [0.69 - 1.25] | 0.629 |
|  | N = 43 | | N = 33 | | N = 21 | |
| GCIP^†^ | **0.71**  **[0.33 - 1.54]** | **0.389** | **0.89**  **[0.41 - 1.94]** | **0.775** | **1.07**  **[0.47 - 2.47]** | **0.865** |
| Age^†^ | 1.05  [1.02 - 1.09] | 0.003* | 1.06  [1.02 - 1.09] | 0.002* | 1.06  [1.02 - 1.09] | 0.002 |
| Disease duration^†^ | 0.98  [0.91 - 1.05] | 0.521 | 0.97  [0.70 - 1.27] | 0.361 | 0.97  [0.90 - 1.03] | 0.303 |
| DMT: yes^‡^ | 1.90  [0.56 - 6.48] | 0.306 | 1.76  [0.52 - 5.94] | 0.360 | 1.69  [0.50 - 5.66] | 0.396 |
| EDSS^†^ | 0.95  [0.70 - 1.28] | 0.735 | 0.94  [0.70 - 1.27] | 0.676 | 0.93  [0.69 - 1.26] | 0.657 |

*Associations of pRNFL and GCIP age-adjusted Z-scores below -1, -1.5 and -2 with sustained PIRA for Munich. Multivariable Cox proportional hazard models. Dependent variable: Sustained PIRA (progression independent of relapse activity). Independent variable: pRNFL (peripapillary retinal nerve) or GCIP (GCIP = ganglion cell-inner plexiform layer) as age-adjusted Z-scores. Participants dichotomized according to Z-scores into thicker (> -1, -1.5 and -2) and thinner (≤ -1, -1.5 and -2) groups. Covariables: Age, disease duration since first clinical attack, DMT (disease modifying therapy, dichotomized as yes or no) and EDSS (Expanded Disability Status Scale). aHR = adjusted hazard ratio, CI = confidence interval. † Time-independent variable, ‡ Time-dependent variable. * Statistical significance p < 0.05*

# **Supplementary Table S3. PwRRMS without vs with sustained PIRA**

|  | | **Without sustained PIRA during follow-up**  **(n = 155)** | **With sustained PIRA during follow-up**  **(n = 65)** | **P** |
| --- | --- | --- | --- | --- |
| **Demographic data** | |  |  |  |
| Age (years), mean ± SD | | 36.7 ± 9.6 | 41.4 ± 9.9 | 0.001* |
| Sex, n (%) | Female | 90 (58.1) | 40 (61.5) | 0.655 |
|  | Male | 65 (41.9) | 25 (38.5) |  |
| Ethnicity, n (%) | Caucasian | 152 (98.1) | 64 (98.5) | 0.756 |
|  | Other | 3 (1.9) | 1 (1.5) |  |
| **Clinical data** | |  |  |  |
| Disease duration since first clinical attack (years), median [IQR] | | 3.5 [0.6 - 8.6] | 4.2 [1.9 - 7.6] | 0.409 |
| DMT, n (%) | No DMT | 41 (26.5) | 15 (23.1) | 0.894 |
|  | Low/Intermediate efficacy | 69 (44.5) | 30 (46.2) |  |
|  | High efficacy | 45 (29.0) | 20 (30.8) |  |
| EDSS (score), median [IQR] | | 1.5 [1.0 - 2.0] | 1.5 [1.0 - 2.5] | 0.444 |
| T25FW (seconds), median [IQR] | | 4.2 [3.7 - 4.7] | 4.1 [3.5 - 4.8] | 0.907 |
| 9HPT dominant hand (seconds), median [IQR] | | 17.7 [16.2 - 19.9] | 18.1 [16.4 - 21.2] | 0.235 |
| 9HPT non-dominant hand (seconds), median [IQR] | | 18.8 [17.1 - 21.0] | 19.5 [18.0 - 21.6] | 0.238 |
| SDMT (score), mean ± SD | | 60.8 ± 13.4 | 60.7 ± 13.0 | 0.980 |
| monocular LCLA (letters), median [IQR] | | 45.5 [42.0 - 48.0] | 41.0 [28.0 - 46.0] | 0.002* |
| binocular LCLA (letters), median [IQR] | | 50.0 [46.0 - 53.0] | 47.0 [42.0 - 48.0] | 0.003* |
| **Retinal layer thickness** | |  |  |  |
| pRNFL (µm), mean ± SD | | 98.9 ± 11.4 | 98.2 ± 12.1 | 0.667 |
| GCIP (µm), mean ± SD | | 70.1 ± 6.7 | 68.6 ± 6.6 | 0.127 |
| pRNFL Z-score, mean ± SD | | -0.32 ± 1.43 | -0.36 ± 1.50 | 0.854 |
| GCIP Z-score, mean ± SD | | -0.41 ± 1.54 | -0.64 ± 1.45 | 0.315 |
| **Follow-up** | |  |  |  |
| Number of visits, median [IQR] | | 6.0 [4.0 - 7.0] | 6.0 [5.0 - 8.0] | 0.169 |
| Follow-up duration (years), median [IQR] | | 6.0 [4.0 - 7.1] | 6.8 [4.9 - 8.0] | 0.065 |

*Baseline characteristics of pwRRMS with or without sustained PIRA during the follow-up. DMT = disease modifying therapy, EDSS = Expanded Disability Status Scale, T25FW = Timed 25-Foot Walk Test, 9HPT = 9-Hole Peg Test, SDMT = Symbol Digit Modalities Test, LCLA = Low Contrast Letter Acuity, pRNFL = peripapillary retinal nerve fiber layer, GCIP = combined ganglion cell and inner plexiform layer, SD = standard deviation, IQR = interquartile range. Retinal layer thickness only of eyes without history of optic neuritis. * Statistical significance p < 0.05*

# **Supplementary Table S4. Exploratory analyses stratified by disease duration**

|  | Univariable | | Multivariable | |
| --- | --- | --- | --- | --- |
|  | HR [95% CI] | p Value | aHR [95% CI] | p Value |
| ≤1 year (n = 57) |  |  |  |  |
| pRNFL Z-score continuous^†^ | **1.47 [0.93 - 2.32]** | **0.100** | **1.28 [0.88 - 1.88]** | **0.202** |
| Age^†^ |  |  | 1.11 [1.04 - 1.19] | 0.003* |
| DMT: yes^‡^ |  |  | 0.77 [0.23 - 2.54] | 0.668 |
| EDSS^†^ |  |  | 1.03 [0.50 - 2.15] | 0.927 |
| GCIP Z-score continuous^†^ | **1.29 [0.78 - 2.14]** | **0.318** | **1.16 [0.77 - 1.73]** | **0.474** |
| Age^†^ |  |  | 1.12 [1.04 - 1.20] | 0.002* |
| DMT: yes^‡^ |  |  | 0.74 [0.22 - 2.43] | 0.619 |
| EDSS^†^ |  |  | 1.04 [0.49 - 2.22] | 0.918 |
| >1 year (n = 162) |  |  |  |  |
| pRNFL Z-score continuous^†^ | **0.93 [0.77 - 1.12]** | **0.439** | **0.93 [0.78 - 1.11]** | **0.408** |
| Age^†^ |  |  | 1.03 [1.00 - 1.06] | 0.089 |
| DMT: yes^‡^ |  |  | 1.52 [0.73 - 3.14] | 0.262 |
| EDSS^†^ |  |  | 1.10 [0.86 - 1.41] | 0.442 |
| GCIP Z-score continuous^†^ | **1.03 [0.88 - 1.20]** | **0.751** | **1.01 [0.86 - 1.19]** | **0.922** |
| Age^†^ |  |  | 1.02 [0.99 - 1.05] | 0.128 |
| DMT: yes^‡^ |  |  | 1.43 [0.69 - 2.99] | 0.336 |
| EDSS^†^ |  |  | 1.09 [0.85 - 1.41] | 0.491 |

*Associations of pRNFL and GCIP age-adjusted Z-scores on a continuous scale with sustained PIRA stratified by disease duration (≤1 year, >1 year). Univariable and multivariable Cox proportional hazard models. Dependent variable: Sustained PIRA (progression independent of relapse activity). Independent variable: pRNFL (peripapillary retinal nerve) or GCIP (GCIP = ganglion cell-inner plexiform layer) as age-adjusted Z-scores on a continuous scale. Covariates: Age, DMT (disease modifying therapy, dichotomized as yes or no) and EDSS (Expanded Disability Status Scale). aHR = adjusted hazard ratio, CI = confidence interval. † Time-independent variable, ‡ Time-dependent variable. * Statistical significance p < 0.05*

# **Supplementary Table S5. Sensitivity analyses by different disability composites**

# **S5a. EDSS**

| Study population (n = 220) | Univariable | | Multivariable | |
| --- | --- | --- | --- | --- |
|  | HR [95% CI] | p Value | aHR [95% CI] | p Value |
| pRNFL Z-score continuous^†^ | **0.96 [0.75 - 1.22]** | **0.713** | **1.01 [0.79 - 1.29]** | **0.936** |
| Age^†^ |  |  | 1.09 [1.04 - 1.14] | <0.001* |
| Disease duration^†^ |  |  | 0.95 [0.88 - 1.03] | 0.210 |
| DMT: yes^‡^ |  |  | 3.00 [0.89 - 10.1] | 0.077 |
| EDSS^†^ |  |  | 0.71 [0.47 - 1.07] | 0.101 |
| GCIP Z-score continuous^†^ | **1.06 [0.85 - 1.31]** | **0.610** | **1.15 [0.90 - 1.46]** | **0.260** |
| Age^†^ |  |  | 1.10 [1.05 - 1.15] | <0.001* |
| Disease duration^†^ |  |  | 0.94 [0.87 - 1.02] | 0.155 |
| DMT: yes^‡^ |  |  | 2.67 [0.79 - 9.07] | 0.115 |
| EDSS^†^ |  |  | 0.68 [0.44 - 1.04] | 0.072 |

*Associations of pRNFL and GCIP age-adjusted Z-scores on a continuous scale with sustained PIRA for the study population. Univariable and multivariable Cox proportional hazard models. Dependent variable: Sustained PIRA (progression independent of relapse activity). Independent variable: pRNFL (peripapillary retinal nerve) or GCIP (GCIP = ganglion cell-inner plexiform layer) as age-adjusted Z-scores on a continuous scale. Covariates: Age, disease duration since first clinical attack, DMT (disease modifying therapy, dichotomized as yes or no) and EDSS (Expanded Disability Status Scale). aHR = adjusted hazard ratio, CI = confidence interval. † Time-independent variable, ‡ Time-dependent variable. * Statistical significance p < 0.05*

# **S5b. EDSS-Plus (EDSS or T25FW or 9HPT)**

| Study population (n = 220) | Univariable | | Multivariable | |
| --- | --- | --- | --- | --- |
|  | HR [95% CI] | p Value | aHR [95% CI] | p Value |
| pRNFL Z-score continuous^†^ | **1.00 [0.84 - 1.20]** | **0.967** | **1.02 [0.85 - 1.22]** | **0.834** |
| Age^†^ |  |  | 1.05 [1.02 - 1.08] | 0.002* |
| Disease duration^†^ |  |  | 0.98 [0.93 - 1.02] | 0.304 |
| DMT: yes^‡^ |  |  | 1.66 [0.82 - 3.36] | 0.158 |
| EDSS^†^ |  |  | 1.00 [0.77 - 1.30] | 0.986 |
| GCIP Z-score continuous^†^ | **1.06 [0.90 - 1.24]** | **0.490** | **1.07 [0.90 - 1.27]** | **0.439** |
| Age^†^ |  |  | 1.05 [1.02 - 1.08] | 0.003* |
| Disease duration^†^ |  |  | 0.97 [0.93 - 1.02] | 0.301 |
| DMT: yes^‡^ |  |  | 1.56 [0.77 - 3.18] | 0.221 |
| EDSS^†^ |  |  | 0.98 [0.75 - 1.28] | 0.898 |

*Associations of pRNFL and GCIP age-adjusted Z-scores on a continuous scale with sustained PIRA for the study population. Univariable and multivariable Cox proportional hazard models. Dependent variable: Sustained PIRA (progression independent of relapse activity). Independent variable: pRNFL (peripapillary retinal nerve) or GCIP (GCIP = ganglion cell-inner plexiform layer) as age-adjusted Z-scores on a continuous scale. Covariates: Age, disease duration since first clinical attack, DMT (disease modifying therapy, dichotomized as yes or no) and EDSS (Expanded Disability Status Scale). aHR = adjusted hazard ratio, CI = confidence interval. † Time-independent variable, ‡ Time-dependent variable. * Statistical significance p < 0.05*
